# Recovery of Memory B-cell Subsets and Persistence of Antibodies in Convalescent COVID-19 Patients

**DOI:** 10.4269/ajtmh.21-0883

**Published:** 2021-09-27

**Authors:** Anuradha Rajamanickam, Nathella Pavan Kumar, Arul Nancy P, Nandhini Selvaraj, Saravanan Munisankar, Rachel Mariam Renji, Vijayalakshmi V., Manoj Murhekar, Jeromie Wesley Vivian Thangaraj, Muthusamy Santhosh Kumar, C. P. Girish Kumar, Tarun Bhatnagar, Manickam Ponnaiah, R. Sabarinathan, V. Saravana Kumar, Subash Babu

**Affiliations:** ^1^ ICER-ICMR-NIRT-International Center for Excellence in Research, Chennai, Tamil Nadu, India;; ^2^ Immunology-ICMR-National Institute for Research in Tuberculosis, Chennai, Tamil Nadu, India;; ^3^ ICMR-National Institute of Epidemiology, Chennai, Tamil Nadu, India

## Abstract

It is essential to examine the longevity of the defensive immune response engendered by severe acute respiratory syndrome coronavirus-2 (SARS-CoV-2) infection. We examined the SARS-CoV-2-specific antibody responses and *ex vivo* memory B-cell subsets in seven groups of individuals with COVID-19 classified based on days since reverse-transcription polymerase chain reaction confirmation of SARS-CoV-2 infection. Our data showed that the levels of IgG and neutralizing antibodies started increasing from days 15 to 30 to days 61 to 90, and plateaued thereafter. The frequencies of naive B cells and atypical memory B cells decreased from days 15 to 30 to days 61 to 90, and plateaued thereafter. In contrast, the frequencies of immature B cells, classical memory B cells, activated memory B cells, and plasma cells increased from days 15 to 30 to days 61 to 90, and plateaued thereafter. Patients with severe COVID-19 exhibited increased frequencies of naive cells, atypical memory B cells, and activated memory B cells, and lower frequencies of immature B cells, central memory B cells, and plasma cells when compared with patients with mild COVID-19. Therefore, our data suggest modifications in memory B-cell subset frequencies and persistence of humoral immunity in convalescent individuals with COVID-19.

## INTRODUCTION

Severe acute respiratory syndrome coronavirus 2 (SARS-CoV-2) infection is a cause of COVID-19.^1^ Recent studies reported that individuals with COVID-19 could experience various symptoms, including fever, myalgia, fatigue, fibrotic lung disease, and pulmonary vascular disease^2^^,^^3^ and the spectrum of disease varies from asymptomatic disease or mild symptoms to severe pneumonia, acute respiratory distress syndrome (ARDS) and death.^4^

Humoral and cellular immune responses have a vital role in controlling viral infections.^5^^,^^6^ The human memory B-cell response is believed to be long-standing in viral infections.^7^ In short-term studies of COVID-19, data suggest that seroconversion occurs at approximately 2 to 3 weeks after the onset of disease,^8^ and IgM titers start declining considerably before the IgG titers.^9^ Numerous reports stated that after SARS-CoV-2 infection, SARS-CoV-2 antigen-specific responses could persist for several months.^10^^–^^12^ In contrast, memory responses to respiratory syncytial virus decrease over the course of this timeframe.^13^^,^^14^ The memory B-cell response to SARS-CoV-2 progresses for 1.3 months and 6.2 months after infection in a manner that is consistent with antigen persistence.^15^ It has been shown that SARS-CoV-2-specific IgA serum concentration declined 1 month after the onset of symptoms; however, neutralizing IgA persists from days 49 to 73 after symptoms.^16^ The persistence of memory B-cell subsets has been reported by different studies, but the data are still not completely clear.^11^ In the present study, we studied humoral immune responses using a cross-sectional study of seven groups of individuals with COVID-19 classified based on the number of days since reverse-transcription polymerase chain reaction (RT-PCR) confirmation of SARS-CoV-2 infection. Our data provide evidence of dynamic alterations in memory B-cell subsets and long-term persistence of antibodies in COVID-19.

## MATERIALS AND METHODS

### Ethics statement.

The study was approved by the Ethics Committees of ICMR-NIRT (NIRT-I no: 2020047) and ICMR-NIE (NIE/IHEC/202008-01). Informed written consent was obtained from all participants. All methods were performed in accordance with the relevant institutional ethical committee guidelines.

### Study population.

Individuals with acute COVID-19 (15–30 days from RT-PCR confirmation, *N* = 46) and convalescent individuals with COVID-19 (classified by days after infection as follows: 31–60, *N* = 33; 61–90, *N* = 38; 91–120, *N* = 34; 121–150, *N* = 32; 151–180, *N* = 37; and > 180, *N* = 40) residing in Chennai and Tiruvallur were enrolled in the study between November 2020 and December 2020 after providing informed consent from the enrolled study individuals. Those with active COVID-19 infection undergoing home isolation and recovered COVID-19 patients within 0 to 15 days of RT-PCR confirmation were excluded from the study. The patients age ranged between 18 and 75 years. COVID-19 was confirmed by RT-PCR in government-approved laboratories. In brief, nasopharyngeal swabs and oropharyngeal (throat) swabs from individuals suspected of having COVID-19 were obtained by the healthcare provider. RNA isolated and purified from specimens was reverse-transcribed to cDNA and amplified. Thermocycling conditions comprised 30 min at 48°C for RT, 10 min at 95°C for activation of the DNA polymerase, and 45 cycles of 15 s at 95°C and 1 min at 60°C. Fluorescence measurements were performed and the threshold cycle (Ct) value for each sample was estimated by determining the point at which fluorescence surpassed a threshold limit set at the mean plus 10 standard deviations beyond the baseline. A test result was calculated as positive if two or more of the SARS genomic targets exhibited positive results (Ct < 45 cycles) and all positive and negative control reactions were in the accepted range. Those individuals who did not experience any symptoms during the entire course of illness were considered asymptomatic, and those who required supplemental oxygen support therapy or those who were admitted to the ICU for oxygen support were considered severely ill. The others were classified as having mild illness.

### Hematology and ex vivo phenotyping.

Hematology was performed for all individuals using the Act-5 Diff hematology analyzer (Beckman Coulter, Brea, CA). Demographic details and other clinical parameters are shown in Table 1. All antibodies used in the study were from BD Biosciences (San Jose, CA), BD Pharmingen (San Diego, CA), eBiosciences (San Diego, CA), or R&D Systems (Minneapolis, MN). Whole blood was used for ex vivo phenotyping, which was performed for all individuals. Briefly, a cocktail of monoclonal antibodies specific for various immune cell types was added to 250-µL aliquots of whole blood. B-cell phenotyping was performed using antibodies directed against CD45-PerCP, CD19-Pacific Blue, CD27-APC-Cy7, CD21-FITC, CD20-PE, and CD10-APC. Naive B cells were classified as CD45^+^ CD19^+^ CD21^+^ CD27^−^; classical memory B cells were classified as CD45^+^ CD19^+^ CD21^+^ CD27^+^; activated memory B cells were classified as CD45^+^ CD19^+^ CD21^-^ CD27^+^; atypical memory B cells were classified as CD45^+^ CD19^+^ CD21^−^CD27^−^; immature B cells were classified as CD45^+^ CD19^+^ CD21^+^ CD10^+^; and plasma cells were classified as CD45^+^ CD19^+^ CD21^−^ CD20^−17^. After 30 min of incubation at room temperature, erythrocytes were lysed using 2 mL of FACS lysing solution (BD Biosciences Pharmingen), and cells were washed twice with 2 mL of 1X phosphate-buffered saline and suspended in 200 µL of phosphate-buffered saline (Lonza, Walkersville, MD). Eight-color flow cytometry was performed on a FACS Canto II flow cytometer with FACSDIVA software (version 6; Becton Dickinson). The gating was set by forward and side scatter, and 100,000 gated events were acquired. Data were collected and analyzed using FLOW JO software (TreeStar, Ashland, OR). Leukocytes were gated using CD45 expression versus side scatter. We used isotype controls to gate all subsets.

**Table 1:** Demographics and clinical parameters of the study population

Days after RT-PCR confirmation of disease	15–30 days	31–60 days	61–90 days	91–120 days	121–150 days	151–180 days	More than 180 days
Subjects enrolled	*N* = 46	*N* = 33	*N* = 38	*N* = 34	*N* = 32	*N* = 37	*N* = 40
Median age (range)	41.5 (18–70)	36 (25–68)	45 (19–59)	45 (21–69)	45.5 (27–59)	42 (23–58)	38.5 (21–78)
Sex (male/female)	27/19	17/18	22/15	22/12	14/18	23/16	26/14
Fever, n (%)	29 (67)	22 (65)	28 (74)	23 (74)	25 (83)	23 (72)	17 (47)
Chills, n (%)	9 (21)	5 (15)	2 (5)	7 (22)	4 (13)	1 (3)	3 (8)
Cough, n (%)	21 (49)	20 (59)	14 (37)	15 (48)	14 (47)	17 (53)	12 (33)
Sore throat, n (%)	21 (49)	12 (35)	11 (29)	12 (38)	10 (33)	16 (50)	13 (36)
Runny nose, n (%)	7 (16)	6 (18)	5 (13)	0	3 (10)	6 (19)	5 (14)
Taste loss, n (%)	24 (55)	14 (41)	17 (44)	12 (39)	11 (37)	20 (63)	12 (33)
Smell loss, n (%)	21 (49)	14 (41)	21 (55)	9 (29)	11 (37)	16 (50)	10 (28)
Muscle aches, n (%)	23 (53)	20 (59)	29 (76)	15 (48)	18 (60)	21 (66)	13 (36)
Joint pain, n (%)	21 (49)	18 (53)	20 (53)	10 (32)	18 (60)	14 (44)	9 (25)
Abdominal pain, n (%)	3 (7)	3 (9)	4 (11)	2 (6.5)	3 (10)	2 (7)	3 (8)
Vomit, n (%)	3 (7)	4 (12)	5 (13)	4 (13)	3 (10)	5 (16)	3 (8)
Diarrhea, n (%)	10 (23)	5 (15)	4 (11)	4 (13)	6 (30)	5 (16)	2 (6)
Seizures, n (%)	0	1 (3)	0	0	0	0	0
Hypertension, n (%)	11 (26)	7 (21)	7 (18)	7 (23)	9 (30)	9 (28)	8 (22)
Diabetes, n (%)	8 (19)	7 (21)	11 (30)	9 (29)	11 (37)	8 (25)	7 (19)
Asthma, n (%)	2 (5)	2 (6)	1 (3)	1 (3)	0	1 (3)	0
Chronic kidney disease, n (%)	0	0	0	0	1 (3)	0	1 (3)
Neurological symptoms, n (%)	0	0	2 (5)	0	0	0	0
Heart symptoms, n (%)	1 (6)	2 (3)	1 (3)	0	0	1 (3)	0
Rheumatic fever, n (%)	0	0	1 (3)	0	0	1 (3)	0
Corticosteroids, n (%)	4 (9)	3 (9)	2 (5)	3 (10)	1 (3)	1 (3)	0
Antiviral drugs, n (%)	4 (9)	5 (15)	2 (5)	4 (13)	0	0	0

RT-PCR = reverse-transcription polymerase chain reaction.

### Measurements of SARS‐CoV‐2 IgA, IgM, and IgG.

The SARS-CoV-2 serology was measured by an iFLASH 1800 chemiluminescent immunoassay from Shenzhen YHLO Biotech, which measures IgM and IgG assays against both SARS-CoV-2 S proteins and N proteins. The tests were performed according to the manufacturer’s protocol (Shenzhen YHLO Biotech Co., Ltd.) The results were determined by chemiluminescent reaction as relative light units. IgM and IgG concentrations were obtained using the iFLASH 1800 assay; ≥ 10 AU/mL was defined as positive and < 10.00 AU/mL was considered nonreactive. Nucleocapsid-specific IgA levels were detected using COVID-19 human IgA ELISA kit (Ray Biotech) based on the manufacturer’s protocol.

### Measurement of circulating neutralizing antibodies.

The circulating neutralizing antibody levels in plasma samples were measured using SARS-CoV2 Surrogate Virus Neutralization Test Kit according to the manufacturer’s (GenScript) instructions. The cut-off value for SARS‐CoV2 neutralizing antibody detection, according to the manufacturer, was neutralization ≥ 20%. Values <  20% were considered nonreactive.

### Statistical analysis.

Data analyses were performed using GraphPad PRISM.9 (GraphPad Software, Inc., San Diego, CA). A cross-sectional analysis of the frequency of memory cell subsets and a hematology analysis was performed using the polynomial model for best fit curve (either first-order or second-order model). Geometric means (GM) were used for measurements of central tendency. Statistically significant differences were analyzed using the nonparametric Mann-Whitney *U* test to compare mild and severe cases.

## RESULTS

### Study population characteristics.

The study population demographics and clinical characteristics are provided in Table 1. No significant differences in age or sex were observed between the study groups.

### Expansion of IgG and neutralizing capacity and decreasing IgA frequencies in convalescent COVID-19 individuals over time.

**Figure 1.**
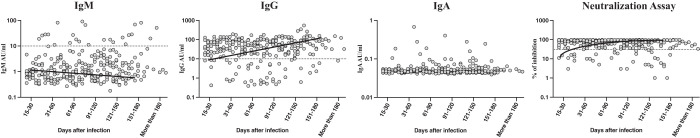
Expansion of IgG and the neutralizing capacity and decrease of the IgA frequencies in convalescent COVID-19 individuals over time. The plasma levels of SARS-CoV2 spike protein-specific IgM and IgG, N protein-specific IgA, and neutralizing antibodies were measured in individuals with acute COVID-19 and convalescent individuals with COVID-19 classified as groups based on the number of days since reverse-transcription polymerase chain reaction confirmation of disease. The levels of antibodies are shown with the preferred model for the best fit curve, and each dot represents single individuals. The thick black line represents the best fit curve.

To estimate the humoral immunity in individuals with acute COVID-19 and convalescent individuals with COVID-19 over time, we examined the plasma levels of SARS-CoV2-specific IgM, IgG, IgA, and neutralizing antibodies in seven groups of individuals with COVID-19. As illustrated in Figure 1, the cross-sectional analysis showed that the IgM levels decreased from days 31 to 60, and then steadily thereafter (first-order model polynomial model fit curve, R = 0.27 by Akaike’s information criterion [AIC]). After 121 days of infection, the IgM levels plateaued. In contrast, the IgG levels (first-order model polynomial model fit curve, R = 0.29 by AIC), IgG levels (second-order model polynomial model fit curve, R = 0.41 by AIC), and neutralizing capacity (first-order model polynomial model fit curve, R = 0.37 by AIC) increased from days 15 to 30 until days 91 to 120 days. After 151 days, all the subsets plateaued. The IgA levels did not show any changes during that time period in convalescent COVID-19 individuals. Therefore, humoral immune response antibody levels are enhanced over time after COVID-19 infection.

### Alterations in frequencies of B-cell subsets in convalescent COVID-19 patients over time.

**Figure 2.**
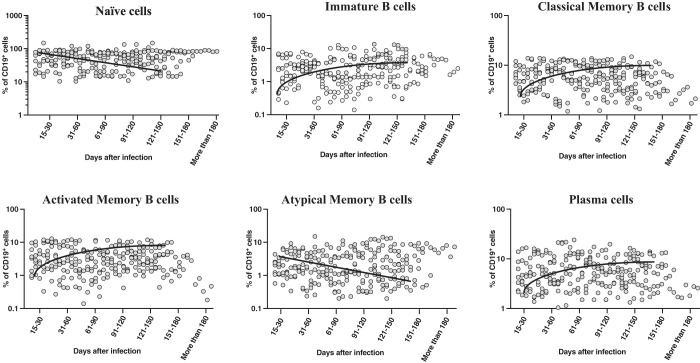
Alterations in the frequencies of B-cell subsets in convalescent individuals with COVID-19 over time. Analysis of B-cell subsets from individuals with acute COVID-19 and convalescent individuals with COVID-19 classified as groups based on the number of days since reverse-transcription polymerase chain reaction confirmation of disease. The frequencies of B-cell subsets are shown with the preferred model for the best fit curve, and each dot represents single individuals. The thick black line represents the best fit curve.

To examine the B-cell phenotype response in acute COVID-19 patients and convalescent COVID-19 patients over time, we assessed the *ex vivo* frequencies of B-cell subsets (naive cells, immature B cells, classical memory B cells, activated memory B cells, atypical memory B cells, and plasma cells) of seven groups of COVID-19 patients. The gating approach is presented in Supplemental Figure 1. As shown in Figure 2, the cross-sectional analysis showed that the frequencies of naive B cells decreased from days 31 to 60, and steadily thereafter (first-order model polynomial model fit curve, R = 0.077 by AIC). After 121 days of infection, the frequencies of naive B cells plateaued. Similarly, atypical memory B cells started declining from days 31 to 60 (first-order model polynomial model fit curve, R = 0.20 by AIC); then, they plateaued after days 121 to 150. In contrast, the frequencies of immature B cells (first-order model polynomial model fit curve, R = 0.41 by AIC), classical memory B cells (second-order model polynomial model fit curve, R = 0.33 by AIC), activated memory B cells (first-order model polynomial model fit curve, R = 0.13 by AIC), and plasma cells (first-order model polynomial model fit curve, R = 0.89 by AIC) increased from days 15 to 30 until days 91 to 120. After 151 days, all the subsets plateaued. Therefore, B-cell subsets frequencies are altered over time after COVID-19 infection.

### Severe COVID-19 disease is associated with altered frequencies of B-cell subsets.

**Figure 3.**
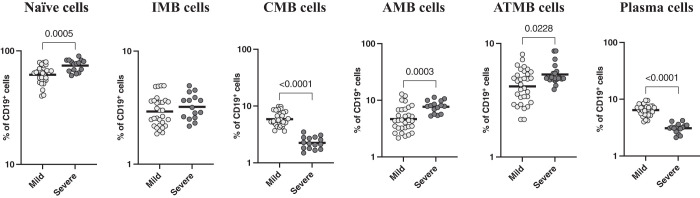
Severe COVID-19 disease is associated with altered frequencies of B-cell subsets. The frequencies of B-cell subsets in individuals with mild (*N* = 30) COVID-19 and severe (*N* = 15) COVID-19 sampled between days 15 and 60 after reverse-transcription polymerase chain reaction confirmation of disease. The data are denoted as scatter plots with each circle denoting a single individual. The Mann-Whitney *U* test was used to calculate *P* values.

To examine the relationship between B-cell subsets and disease severity, we examined the B-cell subsets of individuals with mild and severe COVID-19. As shown in Figure 3, the frequencies of naive cells (GM = 61.62% for mild; GM = 74.27% for severe; *P* = 0.0005), atypical memory B cells (GM = 4.19% for mild; GM = 5.33% for severe; *P* = 0.0228), and activated memory B cells (GM = 4.6% for mild; GM = 7.7% for severe; *P* = 0.0003) were significantly elevated in severe COVID-19 patients compared with mild COVID-19 patients. In contrast, the frequencies of classical memory cells (GM = 5.9% for mild; GM = 2.2% for severe; *P* < 0.0001) and plasma cells (GM = 6.4% for mild; GM = 3.1% for severe; *P* < 0.0001) were significantly lower in severe COVID-19 patients than in mild COVID-19 patients. Therefore, severe COVID-19 disease is linked to altered frequencies of B-cell subsets.

## DISCUSSION

It is crucial to examine the persistence of the defensive immune response induced by SARS-CoV-2- infection. In the present study, we determined both binding and neutralizing antibody levels and performed phenotyping of memory B-cell subsets. It is necessary and important to comprehend the kinetics of SARS-CoV-2-specific antibodies because the results could indicate if the host has generated an efficient humoral immune response. Generally, measurable and high levels of IgM are an indicator of recent infection, whereas IgG levels can be used to identify the incidence of previous infection.^18^ Neutralizing activity has been shown to be linked with defense against reinfection by previous coronaviruses.^19^^,^^20^ A previous study determined that the IgM titers peaked at the time of the acute or initial convalescent phase and then decreased with IgM waning at the end of week 12.^21^ One previous study indicated that SARS-CoV-2-specific IgM levels increased from day 15 after the onset of symptoms and then declined after 35 days after symptom onset.^22^ One recent study reported that the IgM titer decreased at 6 to 7 weeks after symptom onset,^23^ and that IgG antibodies persisted at a high level and could still increase at more than 6 months after symptom onset.^24^ The IgG and neutralization antibody levels were detected at day 14 after infection and showed no decrease in the levels at 3 to 4 months after infection. Another study showed the rapid decrease of SARS-CoV-2-specific antibodies of IgA, IgM, and IgG in convalescent patients at 4 to 14 weeks after discharge.^25^ In agreement with previous studies, the levels of IgM started to decrease from days 31 to 60, and then decreased steadily thereafter. In contrast, IgG and neutralization antibody levels were increased from days 31 to 60 to days 151 to 180 days, and they did not show significant decreases, even at more than 180 days after infection. This is similar to the results of our previously published, larger study that showed the kinetics of IgG and neutralization antibody levels in convalescent individuals.^26^ Collectively, our data indicate that SARS-CoV-2-specific IgG and neutralization antibody responses persist at least until days 151 to 180 after infection, which could have significant importance in terms of reinfection. To our knowledge, our study is one of the first to examine the persistence of IgA antibodies at 6 months after infection.

Memory B cells are important for long-lasting humoral immunity. B-cell subsets can be defined based on CD21 and CD27 marker expressions.^27^ Classical memory B cells, also known as resting memory B cells, last for months to years and can proliferate and differentiate into antibody-producing cells. Activated memory B cells are primed to develop into antibody-secreting plasma cells.^28^ Various studies have shown that chronic viral infections such as hepatitis B, hepatitis C, and HIV exhibit altered circulating frequencies of B-cell subsets.^29^^–^^31^ One recent study indicated that the B-cell memory^17^ compartment is long-standing for at least 6 months in convalescent individuals with COVID-19.^32^ It has been shown that the frequencies of atypical memory B cells significantly decrease and the frequencies of classical memory B cells significantly expand in convalescent individuals.^33^ Recent longitudinal data regarding B-cell sampling after COVID-19 infection demonstrated that B-cell frequencies are stable or increase over time.^10^^,^^11^ Similarly, our data demonstrated that memory B-cell subsets were altered, and that most of the subsets showed an increase at days 15 to 30, and then plateaued after days 151 to 180 after infection. This suggests that B-cell subsets persist after days 151 to 180 of infection. Interestingly, individuals with severe SARS-CoV-2 infection exhibited higher frequencies of atypical memory B cells than individuals with mild SARS-CoV-2 infection.

We also examined the memory B-cell subset distribution in individuals who had mild disease compared with those with severe disease in samples collected during the first 2 months of infection. Activated memory B cells were significantly more abundant in participants with severe infection compared with those with mild infection.^33^ It has been shown that patients with severe COVID-19 exhibit significant heterogeneity in both effector and immature populations. In critically ill COVID-19 patients, extrafollicular activation was strongly associated with the expansion of atypical memory B cells; this expansion was associated with the robust formation of antibody-secreting cells, increased disease activity, and poorer outcomes.^34^ Patients with severe COVID-19 had strong activation of effector B cells than patients with mild disease.^34^ Similarly, our data showed that severe SARS-CoV-2 infection resulted in higher frequencies of atypical memory B cells and activated memory B cells. It is uncertain whether these cells are also functionally impaired in COVID-19.^33^ Augmented frequencies of activated memory B cells could be caused by enhanced immune activation and extrafollicular activation in the severe infection group compared to the mild infection groups.^33^ Our study was limited because we did not study the functional characteristics of these alterations in cellular subsets. Furthermore, we did not explore the persistence of antigen-specific B-cell responses. Finally, the sample collection was cross-sectional and not longitudinal.

In summary, we demonstrated the persistence of humoral immune responses, including both binding and neutralizing antibodies as well as dynamic alterations of B-cell subsets in seven groups of COVID-19 patients. Our data showed that the antibody levels of IgG and neutralizing capacity gradually increased from days 15 to 30 to days 151 to 180 after infection. The alteration of B-cell subsets persists from days 15 to 30 to days 151 to 180 after infection. Our study also offers novel insight into the importance of changes in antibody responses over time and the alteration of B-cell subpopulations over time in COVID-19 patients. These results might aid in the comprehension of B-cell subset dynamics and the identification of more specific concerns related to their clinical effects.

## Supplemental Material


Supplemental materials
