# Supplementary figures and images for: Recovery of Memory B-cell Subsets and Persistence of Antibodies in Convalescent COVID-19 Patients
Source: Am J Trop Med Hyg. 2021 Sep 27;105(5):1255–60. doi: 10.4269/ajtmh.21-0883 (PMC8592221; doi:10.4269/ajtmh.21-0883)

S.Figure.1

Memory B cell subset responses in convalescent COVID-19 subjects

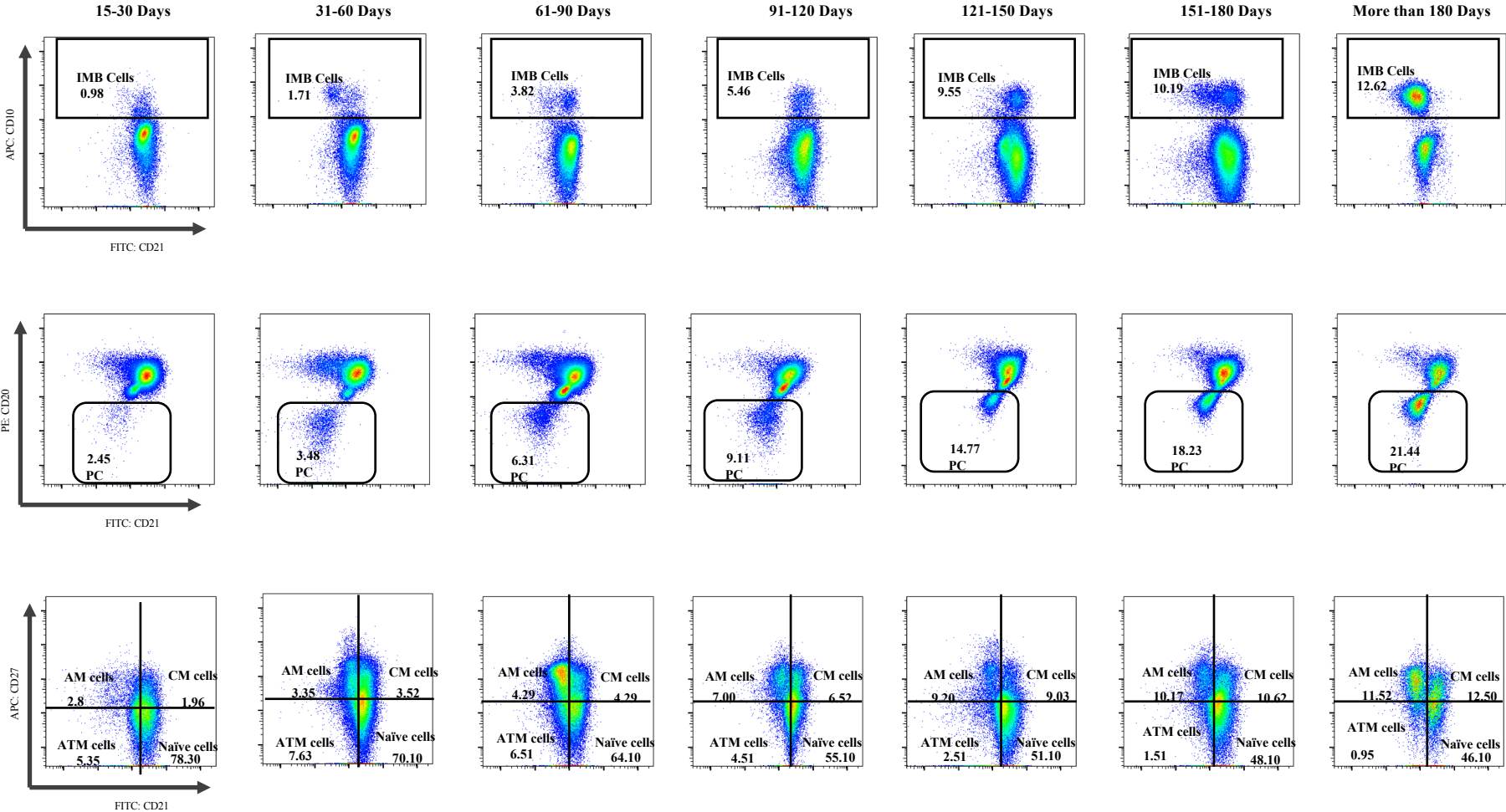

Supplement: Supplementary file 1 [file tpmd210883.SD1.pdf]
